# HLA-B*57:01 screening and hypersensitivity reaction to abacavir between 1999 and 2016 in the OPERA^®^ observational database: a cohort study

**DOI:** 10.1186/s12981-019-0217-3

**Published:** 2019-01-16

**Authors:** Karam Mounzer, Ricky Hsu, Jennifer S. Fusco, Laurence Brunet, Cassidy E. Henegar, Vani Vannappagari, Chris M. Stainsby, Mark S. Shaefer, Leigh Ragone, Gregory P. Fusco

**Affiliations:** 1 Philadelphia FIGHT, 1233 Locust Street, 5th floor, Philadelphia, PA 19107 USA; 2 AIDS Healthcare Foundation, 352 7th Ave., STE 1205, New York, NY 10001 USA; 3 Epividian, Inc, 4505 Emperor Blvd, Suite 220, Durham, NC 27703 USA; 4 ViiV Healthcare, 5 Moore Drive, Research Triangle Park, NC 27709 USA; 5 ViiV Healthcare, 980 Great West Road, Brentford, TW8 9GS UK

**Keywords:** Abacavir, Hypersensitivity reaction, HLA-B*57:01 screening, Cohort, HIV

## Abstract

**Background:**

HLA-B*57:01 screening was added to clinical care guidelines in 2008 to reduce the risk of hypersensitivity reaction from abacavir. The uptake of HLA-B*57:01 screening and incidence of hypersensitivity reaction were assessed in a prospective clinical cohort in the United States to evaluate the effectiveness of this intervention.

**Methods:**

We included all patients initiating an abacavir-containing regimen for the first time in the pre-HLA-B*57:01 screening period (January 1, 1999 to June 14, 2008) or the post-HLA-B*57:01 screening period (June 15, 2008 to January 1, 2016). Yearly incidence of both HLA-B*57:01 screening and physician panel-adjudicated hypersensitivity reactions were calculated and compared.

**Results:**

Of the 9619 patients eligible for the study, 33% initiated abacavir in the pre-screening period and 67% in the post-screening period. Incidence of HLA-B*57:01 screening prior to abacavir initiation increased from 43% in 2009 to 84% in 2015. The incidence of definite or probable hypersensitivity reactions decreased from 1.3% in the pre-screening period to 0.8% in 2009 and further to 0.2% in 2015 in the post-screening period.

**Conclusions:**

Frequency of HLA-B*57:01 screening increased steadily since its first inclusion in treatment guidelines in the United States. This increase in screening was accompanied by a decreasing incidence of definite or probable hypersensitivity reactions over the same period. However, a considerable proportion of patients initiating abacavir were not screened, representing a failed opportunity to prevent hypersensitivity reactions. Where HLA-B*57:01 screening is standard of care, patients should be confirmed negative for this allele before starting abacavir treatment.

## Background

Abacavir, a nucleoside reverse transcriptase inhibitor (NRTI), was approved by the FDA in December 1998. It has since become widely used in combination with other antiretroviral agents to achieve viral suppression and immunologic improvement in patients with HIV infection [1–5]. While abacavir is believed to have a lower propensity for causing mitochondrial toxicity than other NRTIs [6], it has also been linked to potentially fatal hypersensitivity reactions (HSR). Hypersensitivity is an extreme form of adaptive immune response occurring when the immune system reacts inappropriately to certain antigens, and may lead to inflammatory reactions and tissue damage [7]. Abacavir is thought to induce HSR by altering the repertoire of self-peptides presented to T-cells, resulting in an immune response. This is heightened in patients carrying HLA-B*57:01 due to a direct, metabolism-independent and non-covalent interaction of abacavir with HLA-B*57:01 [8–11].

Over 90% of HSR occur in the first 6 weeks following abacavir initiation [12, 13]. Hypersensitivity to abacavir is a multi-organ syndrome characterized by a sign or symptom in two or more of the following categories: (i) fever, (ii) rash, (iii) gastrointestinal (nausea, vomiting, diarrhea or abdominal pain), (iv) constitutional (malaise, fatigue, arthralgia, myalgia), or (v) respiratory (dyspnea, cough, pharyngitis) [14]. Less common signs and symptoms of hypersensitivity include lethargy, myolysis, edema, abnormal chest X-ray, paresthesia, liver failure, renal failure, hypotension, adult respiratory distress syndrome, respiratory failure and death. Reports of anaphylaxis with initial and re-challenge exposure to abacavir have been documented [15–18].

Except for rare fatalities in cases of HSR among patients during their first exposure to abacavir, the symptoms are in general reversed after the discontinuation of abacavir. However, hypersensitivity reaction is much more severe and more likely to be fatal in patients who, after the resolution of initial symptoms, are reintroduced to abacavir. Additionally, there have been reports of individuals who were asymptomatic following initial abacavir use, but developed re-challenge hypersensitivity after use in a subsequent regimen [16, 19].

A genetic link between the risk for abacavir HSR and specific human leukocyte antigen (HLA) alleles HLA-B*57:01 was identified, leading to the introduction of HLA-B*57:01 screening for clinical use in treatment guidelines in the United States on June 15, 2008 [20]. The presence of the HLA-B*57:01 allele detected by HLA-B*57:01 screening has a negative predictive value of 100% and a positive predictive value of 47.9% for immunologically confirmed HSR (i.e. positive result on epicutaneous patch testing 6–10 weeks after clinical diagnosis), as demonstrated by the PREDICT-1 study. However, clinically suspected abacavir HSR were still reported from the HLA-B*57:01 screened group in this study, but at a lower rate (3.4%) compared to the control group (7.8%) [20]. HLA-B*57:01 screening therefore has the potential to eliminate immunologically confirmed HSR and greatly reduce clinically diagnosed HSR incidence [20]. Current guidelines recommend HLA-B*57:01 screening for all patients at the time of ART initiation or modification when an abacavir-containing regimen is considered [21].

The HLA-B*57:01 test was introduced and added to guidelines for clinical care over 10 years ago. The main objective of this study was to describe and compare the annual incidence rate of HLA-B*57:01 screening and HSR before and after June 15, 2008 to assess the use and effectiveness of screening on the occurrence of abacavir HSR in a real-world setting.

## Methods

### Study population

The Observational Pharmaco-Epidemiology Research and Analysis (OPERA^®^) cohort is a clinical cohort including patients from 79 HIV specialty outpatient clinics in 15 US states. For all patients receiving healthcare at a participating site; clinical diagnoses, medications prescribed, and laboratory results are captured prospectively through electronic medical records. Demographic and medical history information are also available. All data reflect routine medical care, with visits and testing scheduled at the discretion of the treating providers. Information captured in the electronic medical records system at each site is retrieved, cleaned, aggregated, and anonymized to maintain patient confidentiality. OPERA^®^ complies with all HIPAA and HITECH requirements and received annual institutional review board approval by Advarra IRB including a waiver of informed consent and authorization for use of protected health information.

All patients initiating an abacavir-containing regimen for the first time between January 1, 1999 and January 1, 2016 were considered for inclusion in the analysis. Included patients were at least 13 years of age with a diagnosis of HIV-1. Additionally, patients were only included if they had at least one clinic visit in the year prior to abacavir initiation to allow baseline characteristic assessment, as well as at least one clinical contact in the year following abacavir initiation to allow for outcome assessment.

### Study design

The observation period began on January 1, 1999 (the date abacavir became widely available) and proceeded until July 31, 2016. Study participants were included in the analysis population through January 1, 2016 to allow a minimum of 6 months of potential follow-up.

The index date for an eligible patient was defined as the first date of the first abacavir-containing regimen ever prescribed to a patient after their first active visit in the OPERA database. Any abacavir-containing regimen was included in the analysis, regardless of formulation or combination with other antiretroviral drugs. The duration of the regimen was defined by discontinuation of abacavir. Changes to other medications within the ART regimen were not considered regimen changes if the patient remained on abacavir.

Patients were observed from their index date until the first of the following censoring events: (i) discontinuation of abacavir, (ii) cessation of continuous clinical activity (i.e. 12 months after the last clinical contact), (iii) death or (iv) study end (July 31, 2016).

Time periods were defined based on the date of abacavir initiation (index date) as (i) pre-HLA-B*57:01 screening period, January 1, 1999 to June 14, 2008, and (ii) post-HLA-B*57:01 screening period, June 15, 2008 to July 31, 2016. Comparisons were made between pre- and post-HLA-B*57:01 screening periods.

### HLA-B*57:01 screening and hypersensitivity reaction to abacavir

All instances of HLA-B*57:01 screening were captured prospectively through electronic medical records. Patients were categorized as screened on the date an HLA-B*57:01 screening was performed, regardless of the results.

Patients with a diagnosis of HSR or symptoms potentially associated with HSR were identified for case adjudication by a panel of physicians. Relevant clinical information was abstracted from the electronic medical record for each of these patients, including symptomology, concurrent medications and diagnoses associated with these symptoms, as well as symptom progression.

A panel of three physicians reviewed each patient’s anonymized clinical information independently to classify them as a definite, probable or possible case, or as not a case. Case determination was based on agreement between two or more of the three physicians. Disagreements on case classification were resolved by discussion among the physicians’ panel.

A definite case of HSR was defined as one of the following within 6 weeks of abacavir initiation; (a) a diagnosis of “hypersensitivity reaction” or “allergic drug reaction” with specific reference to abacavir followed by drug discontinuation within 14 days from the time of onset, (b) two or more symptoms of a hypersensitivity reaction including abdominal pain, allergic reaction, cough, drug reaction, diarrhea, dyspnea, fatigue, fever, flushing, headache, hypersensitivity, malaise, nausea, pharyngitis, rash, or vomiting with complete remission within 14 days from abacavir discontinuation, or (c) a death within 14 days of a diagnosis or a symptom of HSR (cause of death data was not available). A probable case was defined as a single hypersensitivity symptom or other atypical complaints within 6 weeks of initiating abacavir which did not progressively worsen but did not remit until abacavir was discontinued. A possible case of HSR was defined as any of the above criteria greater than 6 weeks after abacavir initiation. Patients who subsequently tolerated abacavir re-challenge were deemed to not be a case.

### Statistical analyses

Data describing clinical and demographic patient characteristics were summarized using medians with interquartile ranges (IQR) for continuous variables and frequencies and proportions for categorical variables. Where applicable, statistical comparisons of patient characteristics by HLA-B*57:01 screening period were made using Pearson’s Chi square or Fisher exact tests for categorical variables and Wilcoxon rank-sum test for continuous variables.

The incidence of HLA-B*57:01 screening and physician-adjudicated HSR was calculated for the pre- and post-HLA-B*57:01 screening periods. Temporal trends were assessed by plotting screening and HSR incidence over calendar years. Kaplan–Meier methods were used to assess time to HSR in the pre- and post-HLA-B*57:01 screening periods. Incidence density was described using incidence rates by year and compared over screening periods using Poisson regression to estimate the incidence rate ratio with 95% confidence intervals.

### Sensitivity analyses

Over time, physicians may have become more adept and confident at identifying HSR. The impact of the differential skill of the physician panel at identifying events in 2016, compared to individual physicians between 1999 and 2016 was therefore assessed. This sensitivity analysis included all diagnoses of HSR and all signs and symptoms consistent with HSR as recorded by the treating physician without regard to adjudication by the panel.

Finally, the main analysis restricted the case definition to events occurring within 6 weeks of abacavir because over 90% of HSR occur within that time frame [12, 13] and late events are more likely to be false-positive events. However, this could have resulted in the exclusion of some true cases. To evaluate the impact of excluding these cases, a sensitivity analysis was undertaken including all events, regardless of their proximity to abacavir initiation.

## Results

### Population characteristics

**Fig. 1:**
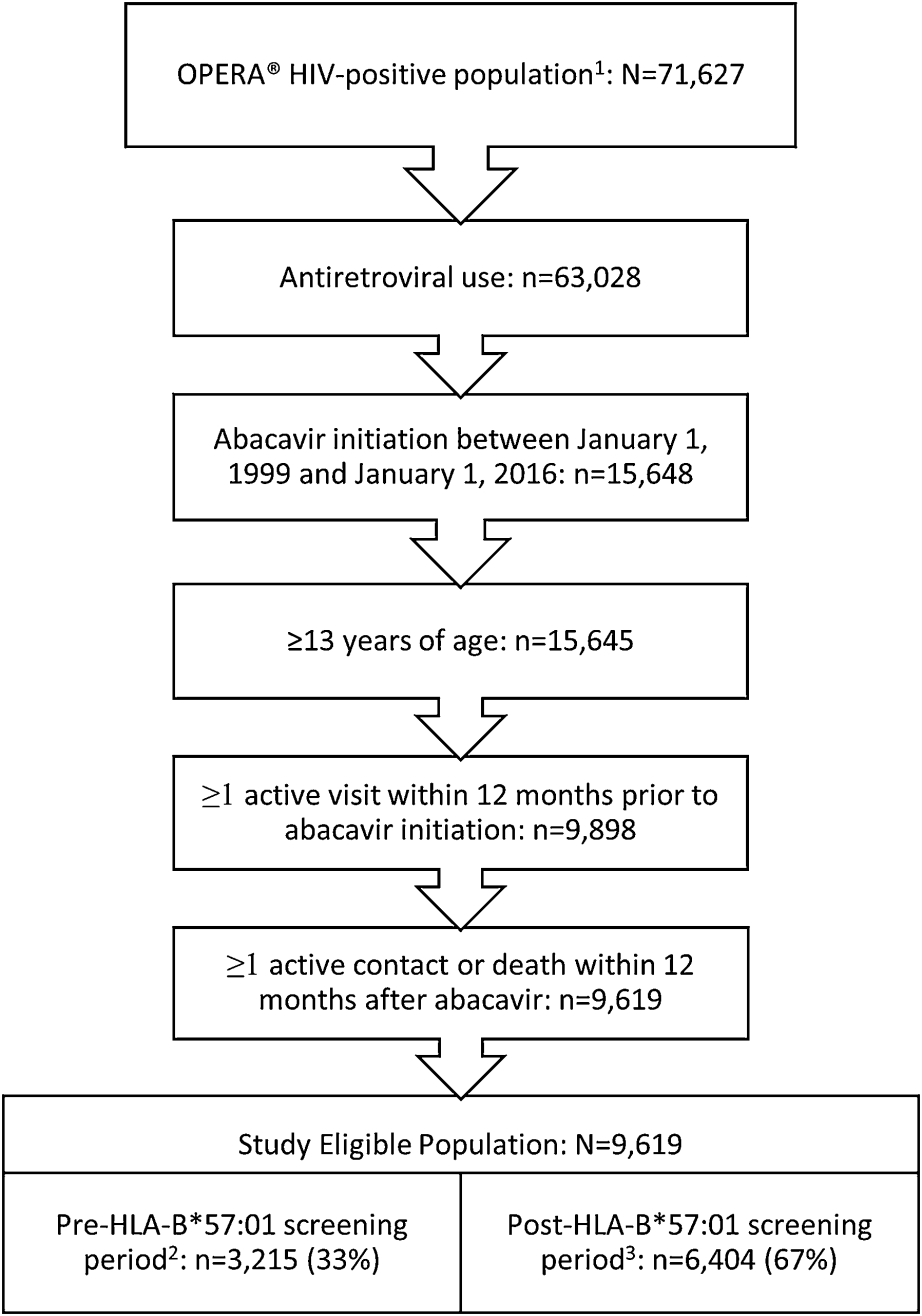
Selection of eligible patients for analysis. ^1^As of July 31, 2016, ^2^Pre-screening period: 01 Jan 1999 to 14 June 2008, ^3^Post-screening period: 15 June 2008 to 31 July 2016

Of the 15,648 HIV-1-positive patients who initiated their first abacavir-containing regimen between January 1, 1999 and January 1, 2016, 9619 were eligible for inclusion in the analysis population (Fig. 1). One-third of the population (n = 3215) initiated abacavir in the pre-HLA-B*57:01 screening period and two-thirds (n = 6404) in the post-screening period.

Demographic and clinical characteristics differed between patients initiating abacavir in the pre- and post-HLA-B*57:01 screening periods (Table 1). Patients in the post-screening period were older and more likely to be female, African American or Hispanic, but less likely to be men who have sex with men (MSM) compared to those in the pre-screening period. Patients initiating abacavir in the post-screening period were also more likely to be ART-naïve, have lower HIV viral loads, have higher CD4 cell counts and less likely to have a history of AIDS defining illness at baseline.

**Table 1:** Demographic and clinical characteristics at index date, by screening period

Characteristic	Pre-screening period^a^ n (%) or median (IQR)	Post-screening period^b^ n (%) or median (IQR)	p-value
N	3215 (33)	6404 (67)	
Age, years	40 (34–46)	44 (34–52)	< 0.0001
Male sex	2759 (86)	5330 (83)	< 0.0001
African American	865 (27)	2375 (37)	< 0.0001
Hispanic ethnicity	615 (19)	1546 (24)	< 0.0001
Men who have sex with men	1969 (61)	3045 (48)	< 0.0001
Antiretroviral therapy naïve	1188 (37)	2752 (43)	< 0.0001
HIV RNA, log10 copies/mL	3.9 (2.2–4.8)	2.0 (1.3–4.5)	< 0.0001
HIV RNA < 50 copies/mL	355 (11)	2625 (41)	< 0.0001
CD4 cell count	274 (142–452)	452 (270–660)	< 0.0001
History of AIDS-defining event	965 (30)	959 (15)	< 0.0001

^a^Pre-screening period: 01 January 1999 to 14 June 2008

^b^Post-screening period: 15 June 2008 to 31 July 2016

### Incidence and trends in HLA-B*57:01 screening and hypersensitivity reactions over time

**Fig. 2:**
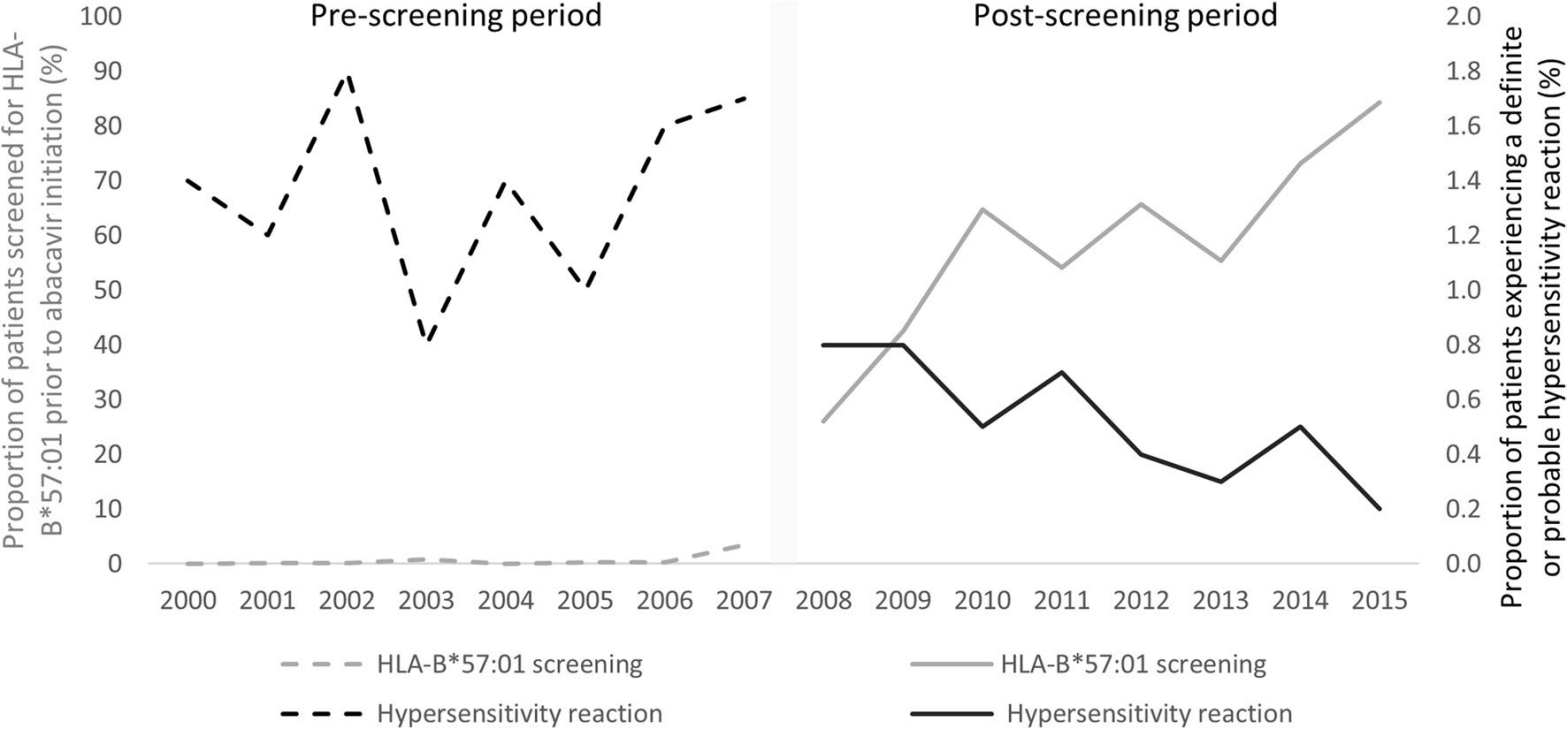
Trends in HLA-B*57:01 screening and physician-adjudicated HSR by calendar year

The proportion of patients screened for HLA-B*57:01 prior to abacavir initiation has increased over time following its introduction to clinical practice in 2008 (Fig. 2). While only 43% of patients were screened prior to abacavir initiation in 2009, 84% were screened in 2015.

Using diagnoses of HSR or signs and symptoms of HSR within the first 6 weeks of exposure to abacavir, 463 (5%) patients were identified for review by a physician panel. Deaths within 14 days of an adjudicated HSR event were rare and did not differ between periods, with one death (0%) pre-screening and six deaths (0.1%) post-screening (p = 0.4370).

Following adjudication, a definite, probable or possible HSR event occurred in 1.6% of patients in the pre-screening period and in 0.6% in the post-screening period (p < 0.0001). Definite, probable or possible HSR occurred a median of 20 days (IQR: 12, 28) after abacavir initiation in both periods (p = 0.9359).

When only definite or probable adjudicated HSR were considered, the overall HSR incidence was further reduced to 1.3% pre-screening and 0.4% post-screening (p < 0.0001). The median time to definite or probable HSR was 17 days (IQR: 10, 27 days) in both periods (p = 0.7028).

Incidence of definite or probable HSR decreased over the study period with a high of 1.8% in 2002 and a low of 0.2% in 2015 (Fig. 2). The incidence remained high during the pre-screening era, varying between 0.8 and 1.8%. However, the incidence of HSR decreased every year following the introduction of HLA-B*57:01 screening, from 0.8% in 2009 to 0.2% in 2015.

### Rates of hypersensitivity reaction and survival time

**Fig. 3:**
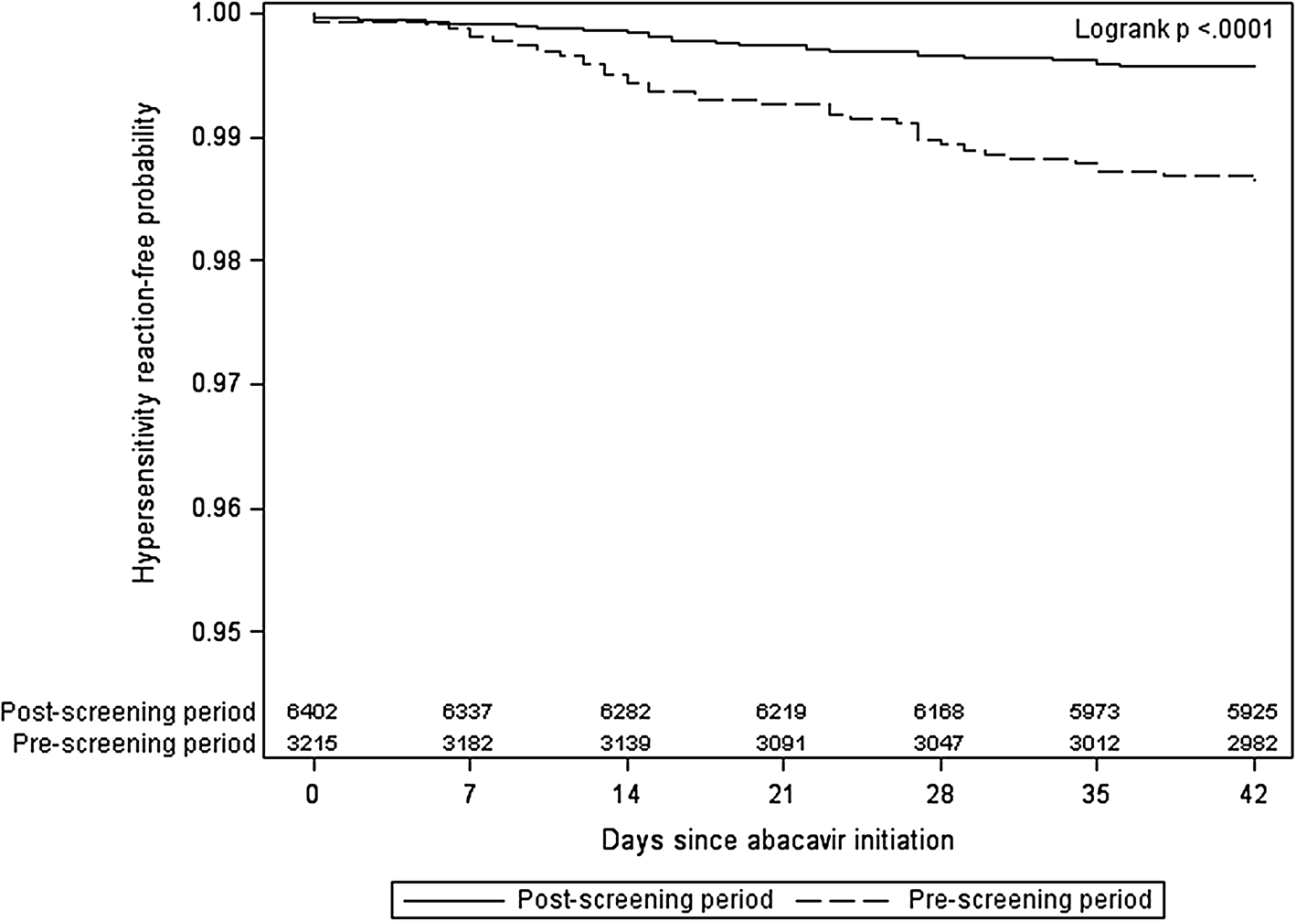
Time to definitive or probable hypersensitivity reaction within 6 weeks of abacavir initiation, by screening period

After 6 weeks (42 days) on abacavir, the overall HSR-free survival probability was 99.3%. The 6-weeks HSR-free survival was 98.7% in the pre-screening period and 99.6% in the post-screening period. The Kaplan–Meier plot for HSR survival revealed statistically significantly different curves by screening period (log-rank p < 0.0001) (Fig. 3).

The incidence density of definite, probable HSR was significantly lower in the post-screening period, with 12 cases per 100,000 person-days of abacavir exposure (IQR: 8, 17) compared to the pre-screening period, with 32 cases per 100,000 person-days (IQR: 23, 44) (Table 2). There was a 69% reduction in incidence rate in the post-screening period compared to the pre-screening period, with an incidence rate ratio of 0.31 (95% CI 0.19, 0.52).

**Table 2:** Incidence density rates of hypersensitivity reactions within 42 days of abacavir initiation, by screening period

Adjudicated hypersensitivity reaction definition	Screening period	Hypersensitivity reaction events	Person-days on abacavir	Incidence rate per 100,000 person-days (95% CI)	Post- vs. pre-screening incidence rate ratio (95% CI)
Any hypersensitivity reaction^a^	Pre-screening^b^	51	129,856	39 (30, 52)	1.00 (Ref)
	Post-screening^c^	37	232,905	16 (12, 22)	0.36 (0.23, 0.55)
Definitive/probable hypersensitivity reaction^d^	Pre-screening^b^	42	129,856	32 (23, 44)	1.00 (Ref)
	Post-screening^c^	27	232,905	12 (8, 17)	0.31 (0.19, 0.52)

^a^Defined as definite, probable or possible hypersensitivity reaction diagnoses

^b^Pre-screening period: 01 January 1999 to 14 June 2008

^c^Post-screening period: 15 June 2008 to 31 July 2016

^d^Defined as definite or probable hypersensitivity reaction diagnoses only

### Sensitivity analyses

In both sensitivity analyses, the same basic trends were observed despite higher incidences of hypersensitivity reactions. First, expanding the event definition to include any diagnosis or sign or symptom of HSR, regardless of physician panel adjudication increased the overall incidence to 7.3% in the pre-screening period and 3.5% in the post-screening period (p < 0.0001).

Many additional HSR events were identified when all physician-adjudicated HSR were included in the second sensitivity analysis, regardless of proximity to abacavir use. Indeed, 22% of all definite or probable HSR identified occurred more than 6 weeks after abacavir initiation. When definite, probable or possible HSR were considered, 53% occurred after 6 weeks. Again, the overall incidence in the pre-screening period was elevated compared to the post-screening period for definite or probable HSR (1.6% vs. 0.5%, p = 0.0005) and for definite, probable or possible HSR (2.8% vs. 1.6%, p < 0.0001).

## Discussion

To the best of our knowledge, this is the first evaluation of HLA*B-57:01 screening practices and changes in HSR incidence in a clinical setting in the United States. In the OPERA^®^ cohort, before the screening test was available, the overall definite or probable HSR incidence was 1.3%. HLA*B-57:01 screening increased steadily after its introduction in June 2008, from 43% screened in 2009 to 84% in 2015. This expansion of screening was accompanied by an important decrease of HSR incidence in the same period from 0.8% in 2009 to 0.2% in 2015.

Estimates of HSR incidence vary greatly based on the study population, clinical practices and case definition used. Clinically, suspected abacavir HSR is diagnosed and managed based on the onset of one symptom from two or more of the following categories: skin rash, fever, GI complaints (nausea, vomiting, diarrhea, abdominal pain), constitutional symptoms (lethargy, malaise, arthralgia, myalgia), or respiratory symptoms (dyspnea, cough, pharyngitis) [22].

A review of clinical trials involving 5332 patients reported an overall HSR incidence of 3.7% [23]. Among these trials were four small studies including fewer than 52 patients each with an HSR incidence of 0%. The incidence reported in the other 20 trials ranged from around 1.5% to 14% [23]. Data from 200,000 patients who received abacavir through clinical trials or by prescription between 1996 and 2000 were described in a large retrospective review in which HSR cases were identified from documented reports of either hypersensitivity/anaphylactic/allergic reaction or symptoms meeting the above clinical diagnosis and reviewed by a physicians’ panel. Among the 30,595 patients receiving abacavir through clinical trials or expanded access program, the incidence of physician-adjudicated definite or probable HSR was 4.3% [13]. However, among the estimated 169,405 patients prescribed abacavir post-marketing, 501 definite or probable spontaneously reported HSR cases were identified. By their nature, data from post-marketing spontaneous reports cannot be as rigorously assessed.

The 1.3% pre-screening period incidence of adjudicated HSR in this study is considerably smaller than the HSR incidences reported among trial participants. However, the incidence estimated in this large clinical cohort is larger than the estimate from the historic review of post-marketing spontaneous reports. These discrepancies can be explained in part by a closer monitoring during trials, and an underestimation of post-marketing events due to reliance on spontaneous reports. The use of electronic medical records in these OPERA analyses allowed a fuller representation of HSR incidence in routine care than spontaneous reports. This, combined with a thorough review of signs and symptoms by a panel of physicians, increased the validity of HSR adjudication and improved the accuracy of the estimates presented here.

While physician panel adjudication of HSR improved the case definition in this study, immunological confirmation of HSR was not available in OPERA^®^, as epicutaneous patch testing for abacavir HSR is a research tool not widely used in clinical practice. Immunological confirmation of HSR greatly reduces its incidence, as demonstrated in a randomized controlled trial assigning patients to either prospective HLA*B-57:01 screening or immediate abacavir initiation with HLA*B-57:01 screening at the end of the 6-week follow-up. Among prospectively screened patients, 3.4% received a clinical HSR diagnosis, although none of the clinical cases were positive with epicutaneous patch testing. Among patients initiating abacavir without undergoing screening, 7.8% were clinically diagnosed, but only 2.7% were immunologically confirmed by epicutaneous patch testing [20].

Although most clinical cohort studies report a higher HSR incidence, a similar trend of considerable reductions in HSR incidence following the introduction of HLA*B-57:01 screening has been previously described in other settings. In Western Australia, HSR incidence dropped from 8% in the pre-screening period (1998 to 2001) to 2% in the post-screening period (2002 to 2005, p = 0.01) [24]. In the UK, a 7.5% HSR incidence was recorded in the pre-screening period (August 2004 to July 2005), compared to 2% in the post-screening period (August 2005 to July 2006, p = 0.03) [25]. While HSR incidences reported in these cohorts are higher than the adjudicated HSR incidence reported here, they are consistent with incidences calculated using diagnosis or symptoms in the sensitivity analysis (7.3% pre-screening and 3.5% post-screening), suggesting a possible overestimation of HSR incidence in routine clinical practice.

In our analysis, the incidence rate of definite or probable HSR was significantly lower in the post-screening period compared to the pre-screening period, with an incidence rate ratio of 0.31 (95% CI 0.19, 0.52). PREDICT-1, a randomized controlled trial reported a similar reduction in clinical HSR diagnoses when patients were prospectively screened rather than initiating abacavir immediately (odds ratio: 0.40, 95% CI 0.25, 0.62) [20].

In this study, the median time to definite or probable HSR (defined as an event occurring within 6 weeks of abacavir initiation) was 17 days, which is longer than the median 8 to 11 days reported in trials [12, 13]. A closer monitoring of patients in the context of a trial could lead to earlier detection of HSR, compared to regular clinical care. However, the median time to HSR reported here is consistent with descriptions of HSR clinical presentation, which has been reported up to 318 days after abacavir initiation [12].

Patients initiating abacavir in the post-screening period were significantly different from those initiating in the pre-screening period. These differences were consistent with the changing HIV epidemic over these time periods, notably as earlier initiation of effective ART results in better control of the infection [26] and longer life expectancy [27]. Because data on cause of death was not available in the OPERA^®^ database, it was impossible to assess HSR-specific mortality. However, death within 14 days following HSR was very rare in this study (0.07%), and consistent with reports of 0.03% mortality in patients who received abacavir in clinical trials [13].

This study made use of the OPERA^®^ database, the largest continuously operating cohort of HIV-infected patients, which includes complete patient health records managed in electronic health record systems and is updated daily. The HIV-infected population in OPERA^®^ receives care at 79 separate locations throughout the United States and represents 7% of all HIV-infected patients linked to care in the United States. Therefore, the incidences of HLA*B-57:01 screening and HSR incidence estimates in this cohort are largely representative of changes in clinical practice in the United States.

However, this study was restricted to patients initiating abacavir. Therefore, HLA*B-57:01 screening among patients who did not initiate abacavir was not assessed, resulting in an underestimation of true HLA*B-57:01 screening uptake. In addition, the HSR adjudication criteria used by the physicians’ panel was different from criteria used in other studies and the current approved product labelling [22]. The retrospective nature of electronic medical records review limited the information available for adjudication. Criteria were therefore developed by a panel of experts to maximize the validity of the adjudication process.

## Conclusions

Expanded HLA*B-57:01 screening in clinical practice has been associated with fewer patients experiencing abacavir-associated hypersensitivity reactions in the OPERA^®^ cohort. However, 16% of patients initiating abacavir in 2015 had not been screened beforehand. This gap indicates a potential for further reducing the risk of HSR by improving clinical practices. Indeed, where HLA-B*57:01 screening is standard of care, patients should be confirmed negative for this allele before starting abacavir treatment, as recommended in clinical guidelines.

## Abbreviations

NRTI: nucleoside reverse transcriptase inhibitor
HSR: hypersensitivity reactions
HLA: human leukocyte antigen
ART: antiretroviral therapy
OPERA^®^: Observational Pharmaco-Epidemiology Research and Analysis
IQR: interquartile range
MSM: men who have sex with men
